# Orthogonal Templating Control of the Crystallisation of Poly(ε-Caprolactone)

**DOI:** 10.3390/polym10030300

**Published:** 2018-03-11

**Authors:** Geoffrey R. Mitchell, Robert H. Olley

**Affiliations:** 1 Centre for Rapid and Sustainable Product Development, Institute Polytechnic of Leiria, 2430-028 Marinha Grande, Portugal; 2 EMLAB, University of Reading, Reading RG6 6AF, UK; hinmeigeng@hotmail.com

**Keywords:** crystallisation, morphology, nanoparticles, shear, flow, orientation, poly(ε-caprolactone), polyvinyl butyral

## Abstract

The crystal growth of poly(ε-caprolactone) can be very effectively directed through the use of small amounts of dibenzylidene sorbitol in conjunction with modest flow fields to yield extremely high levels of the preferred polymer crystal orientation. We show that by introducing small quantities of a terpolymer, based on polyvinyl butyral we can switch the symmetry axis of the final lamellar orientation from parallel to perpendicular to the melt flow direction. During shear flow of the polymer melt, the dibenzylidene sorbitol forms highly extended nanoparticles which adopt a preferred alignment with respect to the flow field and on cooling, polymer crystallisation is directed by these particles. The presence of the terpolymer, based on polyvinyl butyral, limits the aspect ratio of the dibenzylidene sorbitol (DBS) particles, such that the preferred orientation of the particles in the polymer melt changes from parallel to normal to the flow direction. The alignment of lamellar crystals perpendicular to the flow direction has important implications for applications such as scaffolds for tissue engineering and for barrier film properties.

## 1. Introduction

**Scheme 1:**
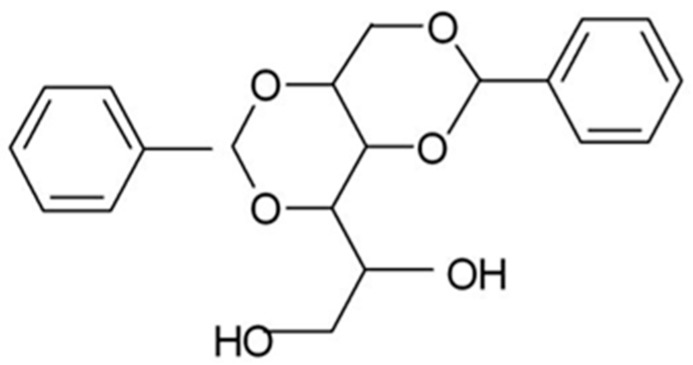
Dibenzylidene Sorbitol (DBS).

Dibenzylidene sorbitol (DBS) (see Scheme 1) [I] is a low molar mass gelator [1,2,3,4]. In a variety of solvents including polymers at very low concentrations, <1% *w*/*w*, it self-assembles into extended crystalline nanofibrils. When DBS is dispersed in a polymer matrix, the application of modest shear flow leads to a macroscopic alignment of the fibrils [5,6,7]. When the matrix is a crystallisable polymer, subsequent crystallisation of the matrix is templated by the DBS nanofibrils [5,6]. In this work, we centre our attention on the control of the templating direction.

The properties of polymer based materials depend on the processing procedures used to shape the object as well as the ingenuity of the molecule maker in terms of the chemical configuration of the polymer chains. In the case of a crystallisable polymer such as poly(ε-caprolactone), the properties of the final product are especially dependent on the arrangement of the chain folded lamellar crystals [8]. This is termed the morphology, they may be aligned as a consequence of a common orientation of the row nuclei, the crystals may be extended in a fibrillary structure providing high modulus [9], or they may be arranged with a variety of orientations within spherulites which is the most common morphology [10] if no particular action is taken. In the case of spherulites, the distribution of crystals may lead to a reduction of the dielectric breakdown strength in the case of insulators [11]. As well as mechanical properties, the distribution of crystals will impact on the optical properties [12], on the degradation rate of the polymer [13] and the permeability of the polymer film to gases such as oxygen [14].

The role of dibenzylidene sorbitol as a nucleating agent for isotactic polypropylene has been known for some time, although the mechanism is less clear. Smith et al., proposed that it stabilized the helical structure through the cleft in the V shaped nature of the molecular structure [15]. Mandelkern has reviewed the nucleation mechanisms and identified the importance of epitaxy but also states “it is not the only one that can be involved” [16]. Lotz and co-workers have identified the strong role of atomistic epitaxy in the nucleation in which there is matching of the crystal structures of the two components [17]. More recently we have explored the use of dibenzylidene sorbitols and some derivatives to direct the crystallization of different polymers. The ubiquity of the process and the similarity to the use of graphene nanoflakes, carbon nanotubes, and other preformed nanoparticles has lead us to the conclusion that the underlying mechanism is most likely to be graphoepitaxy which is a process in which the orienting influence on the growth of a layer on the substrate can be determined by various factors (forces) distinctive of the crystalline lattice [18].

Key factors with regard to dibenzylidene sorbitol enabling the direction of crystallisation are the formation of highly anisotropic nanoparticles which easily align in low shear flow field [7]. The solubility of dibenzylidene sorbitol in a polymer melt is strongly related to the polarity of the solvent as has been shown with low molar mass solvents [7]. Previous work has shown the nucleating effect of dibenzylidene sorbitol in poly(ε-caprolactone) saturates at 3% whereas in polyethylene the effect saturates at ~1% [6].The difference is related to the solubility of the dibenzylidene sorbitol in the polymeric solvent and therefore how many nanoparticles are formed to direct the crystallization.

The work reported here is based on the use of self-assembling nanoparticles rather than engineered nanoparticles which have significant environmental impact issues and will limit the recycling or disposal of objects prepared using this approach. As a compound approved for use in food contact applications DBS has none of the potential hazards of engineered nanoparticles such as carbon nanotubes or metallic particles.

DBS is widely employed as a clarifying agent with isotactic polypropylene where it yields films with a high level of optical transparency for packaging [12]. It is also widely used in cosmetics and small quantities are thus unlikely to have any adverse effect in the environment. In this work, we focus on poly(ε-caprolactone) (PCL) which is a biocompatible and biodegradable polymer widely used for the manufacture of biomedical devices including scaffolds for tissue engineering and drug delivery systems. It is sufficiently stable thermally to be considered for implants in the human body, its low melting point (55–60 °C) has allowed its use in “fantastic plastic” demonstrations in which it is melted in hot water and moulded by hand. Keith et al., reported that the introduction of small quantities of polyvinyl butyral (PVB) could profoundly affect the nucleation of PCL spherulites with major changes in the banding in the spherulites [19]. Use of PVB in conjunction with PCL to produce large spherulites has been exploited to allow detailed studies of the spherulite banding using microbeam wide-angle and small-angle X-ray scattering (WAXS and SAXS) techniques [20,21] and electron tomography [22]. Such studies have shown PCL and PVB to be compatible in the melt. PVB has similar suppressive, although less pronounced, effects on the nucleation in other polyesters [19]. Here we consider a PCL system which contains both the nucleating and directing additive DBS as well as the nucleating suppression agent PVB. We use in-situ time-resolving X-ray scattering techniques to explore the potential consequences of the interactions between these competing additives on the flow and crystallisation of these novel mixtures.

## 2. Materials and Methods

The polyvinyl butyral based terpolymer (commonly abbreviated in industrial practice as PVB) used in this work is a random terpolymer containing ~88% of polyvinyl butyral together with ~0.5% polyvinyl acetate and ~11.5% polyvinyl alcohol units and was obtained from Aldrich (St. Louis, MO, USA). The molecular weight is 90,000 to 120,000 Da. 3% *w*/*w* dibenzylidene sorbitol (Ciba, Basel, Switzerland) and 3% *w*/*w* polyvinyl butyral were dispersed alone and together in PCL (Aldrich, St. Louis, MO, USA) (*MW* = 80,000 Da) by solution blending using butanone as the co-solvent, cooling the solution to room temperature and allowing the butanone to evaporate under the draught of a fume cupboard. Moulded discs for the shear flow experiments were prepared from these mixtures by melt moulding at 80 °C, followed by cooling to 0 °C to allow the PCL to crystallise fully before warming to room temperature.

**Figure 1:**
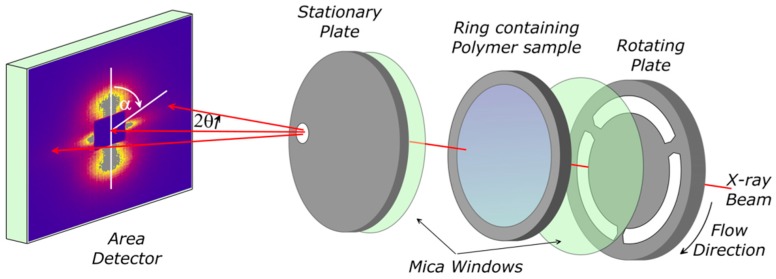
Schematic of X-ray shear cell system used in this work Adapted from [24] (Wangsoub, S.; Oiley, R.H.; Mitchell, G.R. Directed Crystallisation of Poly(ε-caprolactone) using a Low-Molar-Mass Self-Assembled Template. *Macromol. Chem. Phys.* **2005**, *206*, 1826–1839).

Samples of PCL, PCL/DBS, PCL/PVB, and PCL/DBS/PVB were subjected to controlled flow and thermal profiles using a specially designed parallel plate shear flow system [23,24] as shown in Figure 1. The sample was held between two thin mica discs supported on slotted metal plates mounted within a small oven equipped with both electrical heating and refrigerated gas cooling, providing a controlled temperature range from −20 to 300 °C with heating and cooling rates up to 20 °C/min. This shear flow cell system enabled X-ray scattering experiments to be performed during and following controlled shear flow. The slots in the metal plates allowed the X-ray beam to pass through the sample for ~85% of a revolution. Collimation and masks prior to the rotating plate in the beam line were used to minimize the parasitic scattering from the spokes of the rotating plate as they cut the incident beam. An intelligent motor control system ensured that when the rotation was stopped, this was at a rotation angle where the beam was not obscured by a metal spoke.

Time resolved X-ray scattering experiments were performed on the fixed wavelength (λ = 1.54 Å) beam-line 2.1 [25] at the Daresbury Synchrotron Radiation Source (UK). Small-angle X-ray scattering (SAXS) data were recorded using a 2-D RAPID detector which allowed scattering data to be accumulated in the range |Q| ~ 0.01 to 0.2 Å^−1^ (|Q| = 4π sin θ/λ where λ is the incident wavelength and 2θ is the scattering angle) with a data accumulation time of 10 s. The sample to detector distance and the detector pixel size were calibrated in terms of geometry using wet collagen.

Wide-angle X-ray Scattering (WAXS) were obtained at room temperature on static samples using a symmetrical transmission diffractometer equipped with a graphite monochromator and a copper X-ray sealed tube source. The intensity was measured as a function of |Q| and α, the angle between the symmetry axis of the sample and Q, in steps of |∆Q| = 0.02 Å^−1^ and in steps ∆α = 5°.

Specimens for optical microscopy were prepared on glass coverslips in a Metter FP hotstage by melting for 2 min at 150 °C, then cooling rapidly (~30 °C/min) to 60 °C followed by cooling at a rate of 1 °C/min to 30 °C before removing to room temperature. Optical microscopy was performed at room temperature using a Carl Zeiss GFL polarised light microscope.

## 3. Results

**Figure 2:**
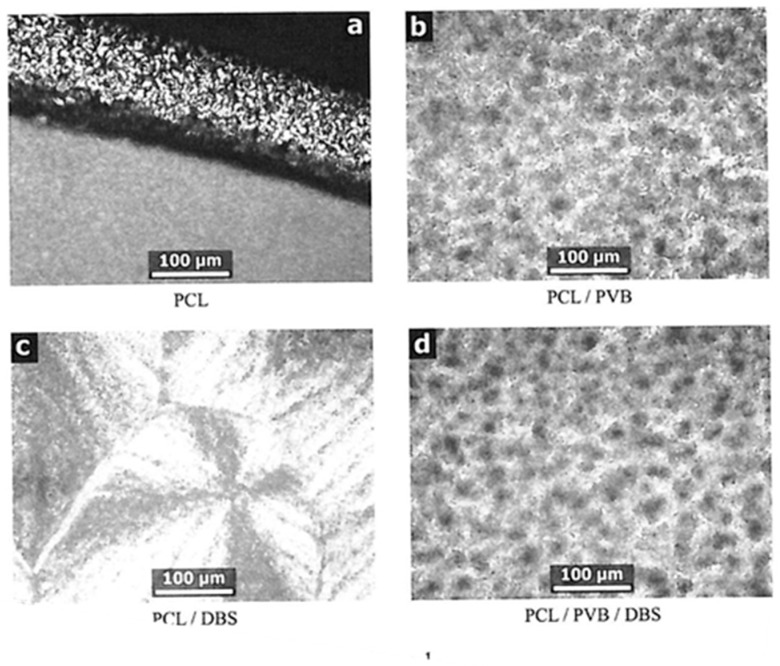
Optical Micrographs obtained in a polarised light microscope at room temperature of samples of (**a**) PCL; (**b**) PCL with 3%PVB; (**c**) PCL with DBS; and (**d**) PCL with 3% PVB and 3% DBS which were crystallised from the melt as described in the text.

Figure 2a shows an optical micrograph with the typical spherulitic morphology for PCL crystallized under quiescent conditions while cooling from 60 to 30 °C at 1 °C/min, after melting at 150 °C. The picture is taken at the edge of a film approximately 0.1 mm thick—such films are used to ensure that the sample is representative of the material as a whole, instead of possibly being dominated by nucleation on the glass slide and cover slip surfaces. The bulk film (lower half of picture) is nucleated heavily so that individual spherulites appear to be of the order of 10 µm or less in diameter. At the top of the picture is the edge of the specimen, where a much thinner film is left as a result of smearing on one of the glass surfaces. Here individual objects are more easily discernible, generally up to 20 µm in diameter, but larger spherulites always tend to form in such situations in many kinds of polymers, so this cannot be taken as representative of the material as a whole. The addition of 3% PVB (Figure 2b) gives rise to a specimen containing much larger spherulites, typically 50 µm in diameter. This is consistent with the earlier observations by Keith et al. [19] that PVB greatly reduced the nucleation density in PCL.

For samples of PCL containing 3% DBS, in-situ SAXS experiments have shown that at 150 °C the DBS will be fully dissolved in the PCL [24]. The PCL/DBS sample (Figure 2c) prepared from a melt at 150 °C as described above gives rise to a much greater nucleation density, such that the individual objects are not at all properly discernible at this magnification. The texture has the appearance of large grainy spherulites. These are made up of small PCL crystals which have crystallised preferentially with respect to fibrils of DBS which crystallised at a higher temperature from solution in molten PCL [24].

The specimen containing 3% DBS and 3% PVB was prepared by melting at 170 °C before being given the same treatment as the previous samples. This resultant micrograph appears to be the same as shown in Figure 2b for the PCL/PVB sample, suggesting either that the PVB is preventing the DBS from crystallising during cooling, or it is completely inhibiting the nucleation effect of the DBS on the PCL. If we repeat the treatment but cooling from a lower melt temperature where the DBS will not be fully dissolved, the morphology is more mixed between that shown in Figure 2a,d suggesting that the PVB has the capacity to suppress or modify the crystallisation of the DBS as well as that of the PCL.

**Figure 3:**
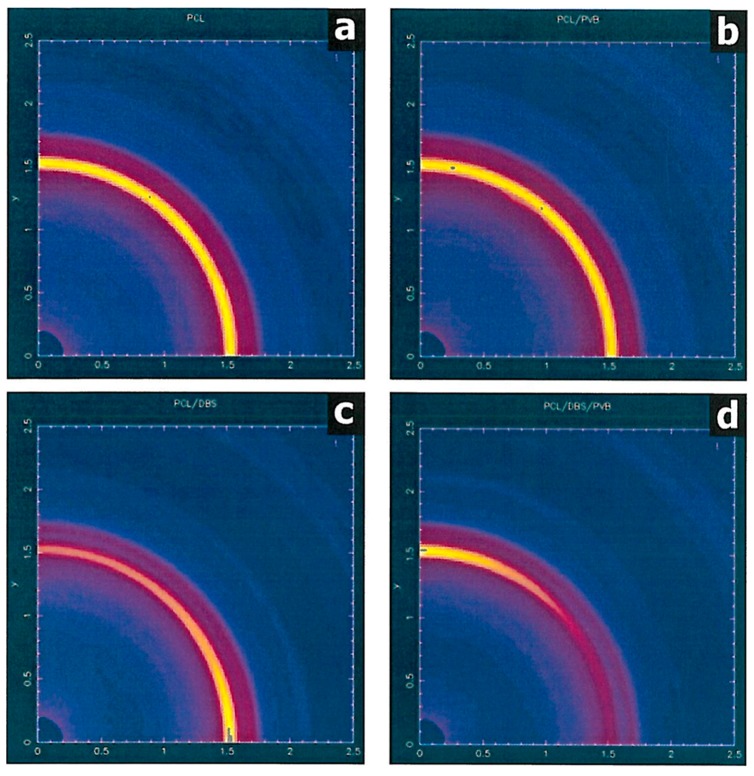
Maps of the wide-angle X-ray scattering (WAXS) intensity obtained for samples of (**a**) PCL; (**b**) PCL with 3%PVB; (**c**) PCL with 3% DBS; and (**d**) PCL with PVB and 3% DBS crystallised from a melt after a shear flow of I0 s^−1^ for 100 s at 80 °C. The melt flow direction is vertical.

Samples of PCL, PCL/DBS, PCL/PVB, and PCL/DBS/PVB were processed using a defined thermal and shear profile which consisted of heating to 80 °C at 20 °C/min, holding for 5 min and then subjected to a shear flow field of 10 s^−1^ for 100 s (shear strain = shear rate x time = 1000 su). After cessation of shearing the sample was cooled to room temperature at rate of 10 °C/min. Figure 3a shows the WAXS intensity map for the PCL sample, which, although it has been crystallised from a sheared melt, nevertheless exhibits an isotropic pattern typical of a semi-crystalline polymer. The pattern exhibits several crystalline rings (seen as arcs), of which by far the strongest is from the (110) planes at |Q| ~1.5 Å^–1^. This is in-line with previous studies and reflects the zero memory of the shear field due to the rapid relaxation of the PCL chains after shearing before crystallisation take place [24]. The corresponding PCL/PVB specimen shows the same isotropic pattern (Figure 3b). The PCL/DBS specimen, on the other hand, shows a considerable level of anisotropy in the WAXS pattern (Figure 3c). The specimen is mounted so that the shear direction is vertical and the 110 reflection is most intense on the equatorial section normal to the flow direction. This indicates that lamellae are aligned normal to the flow direction although there is an ambiguity as the preferential alignment of the c-axis of the PCL crystals parallel to the melt flow. This distribution of intensity is consistent with the model in which the PCL crystals are directed by DBS fibrils which have been previously preferentially aligned parallel to the flow direction in the PCL melt phase [24].

The WAXS pattern for the PCL/PVB/DBS specimen (Figure 3d) is also substantially anisotropic. In contrast to the PCL/DBS sample, the 110 reflection is most intense on the meridional section, indicating an orientation of the PCL lamellae which is orthogonal to that displayed by the PCL/DBS sample.

**Figure 4:**
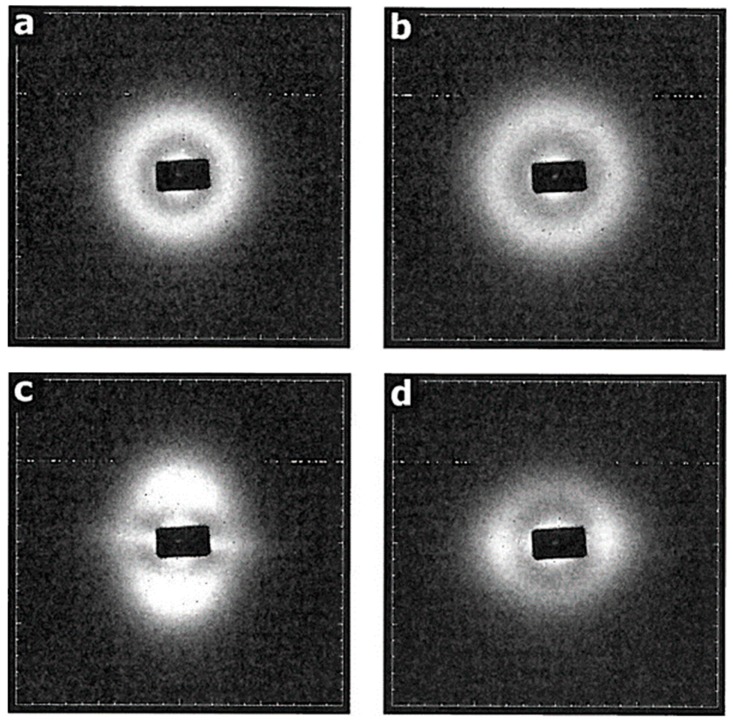
Small-angle X-ray scattering (SAXS) patterns obtained for samples of (**a**) PCL at 20 °C; (**b**) PCL with 3% PVB at 7 °C; (**c**) PCL with DBS at 22 °C and (**d**) PCL with 3% PVB and 3% DBS at 19 °C, all crystallised from a melt after a shear flow of 10 s^−1^ for 100 su at 80 °C. The melt flow direction is vertical. Each pattern is shown for the temperature where the invariant recorded during crystallisation was at its maximum.

Figure 4 shows the SAXS patterns for the four different materials considered here recorded at the point where the invariant (see below) reaches a maximum during the cooling stage of the thermal and shear profile described above.

The PCL sample exhibits a SAXS pattern which is typical for many semi-crystalline polymers [26]. The maximum in intensity at |Q|~0.032 Å^–1^, corresponding to a long period of ~195 Å, arising from the lamellar structure. The constant intensity as a function of the azimuthal angle shows that the lamellae have no preferred orientation with respect to the melt flow direction. A similar pattern is displayed by the PCL/PVB sample (Figure 4b) with the lamellar peak at a |Q| value of ~0.0385 Å^–1^, corresponding to a long period ~165 Å. The PCL/DBS sample exhibits a SAXS pattern (Figure 4c) which is strongly anisotropic. The intensity scattering on the meridional axis indicates a strong preferred orientation of the lamellae normal to what was the melt flow direction (vertical on the page); this is consistent with the WAXS result. The |Q| value for the maximum is ~0.0325 Å^−1^, corresponding to a long period of ~195 Å. This confirms observations made previously that the DBS directs the lamellar growth direction but does not otherwise change the lamellar characteristics [7,26]. There is also a horizontal streak on the equatorial section which was not present in the patterns recorded for the PCL and PCL/PVB samples. This relates to the presence of highly extended DBS fibrils which are ~150 Å in width and 1000 Å or more in length. In striking contrast the PCL/PVB/DBS (Figure 4d) shows a similar pattern but one in which the lamellar peaks are now on the equator indicating that the lamellae are preferentially aligned parallel to the flow direction in accord with the crystal plane orientation information available from the WAXS patterns. Here the |Q| value is ~0.0335 Å^–1^, corresponding to a long period ~190 Å. Examination of the scattering around the beamstop reveals that there is no highly anisotropic equatorial streak as observed in the PCL/DBS sample. In fact, the portion of the scattering appears almost isotropic but there is a substantial level of scattering here as compared to that observed in the patterns for the PCL and PCL/PVB samples. Analysis of this scattering gives a particle radius of ~40 Å diameter which is smaller than that observed for the PCL/DBS system. The lamellar scattering is also noticeably weaker than the equivalent pattern observed for the PCL/DBS.

During the thermal and shear profile for each sample, we recorded SAXS patterns on a time resolving basis with a time cycle of 10 s throughout the processing cycle. This enables us to follow the formation of structure including the crystallisation. For each SAXS pattern we have calculated the so-called invariant Ω [27]:

(1) $$\Omega =\;\int_{\alpha =0}^{\pi /2}\int_{Q=0}^{Q_{max}}{\left|Q\right|}^{2}I\left(\left|Q\right|,\alpha \right)\sin\alpha \;dQd\alpha$$

**Figure 5:**
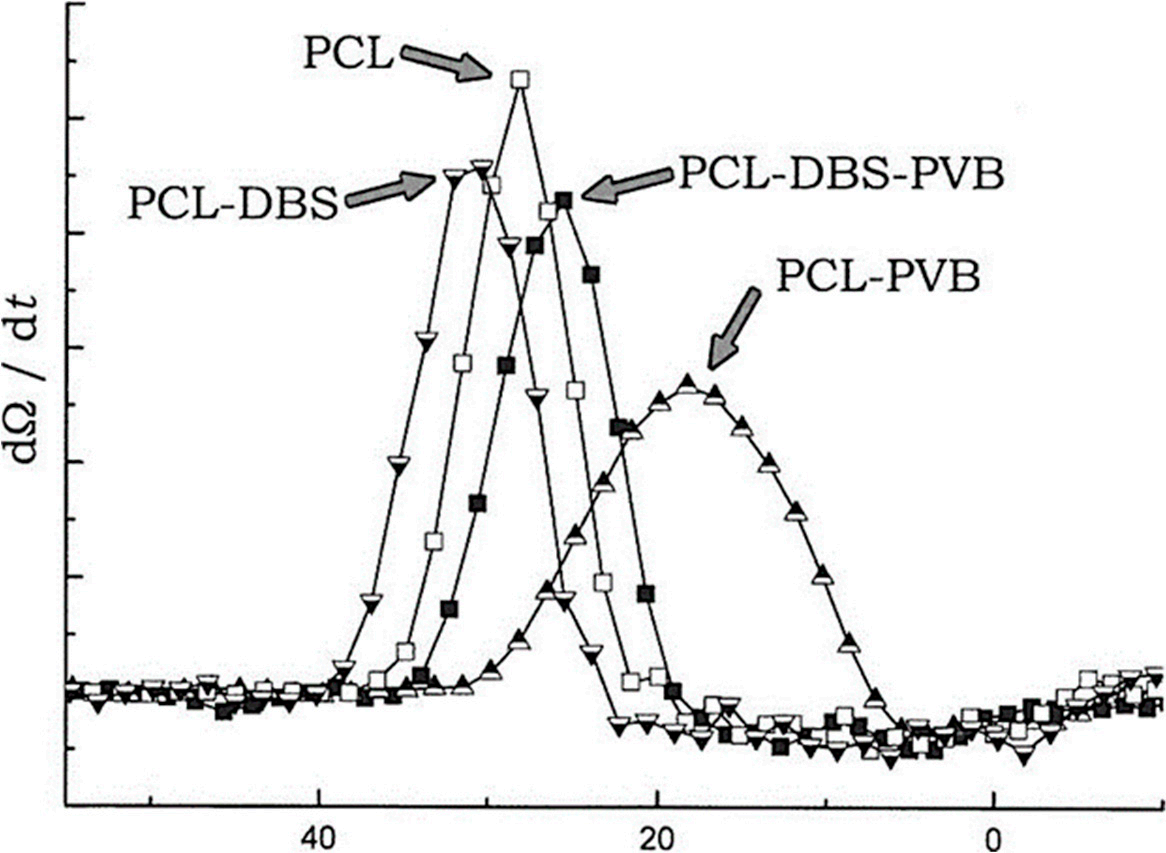
Plots of the derivative of the invariant against the temperature of measurements for each of the four materials considered in this work during the crystallisation stage of the processing cycle described in the text. The uncertainties associated with each data point are horizontally ±1 °C, vertically 2% of the vertical range.

The invariant is related to the volume fraction of crystals, at least in the initial stage of crystallisation, as all other things being equal, the invariant would also go through a maximum when the volume occupied by lamellae reaches 50%, and it will decrease slightly on cooling as the electron densities of crystalline and amorphous material approach each other. We have used the plots of invariant versus temperature to identify the temperature at which the maximum rate of crystallisation occurs. Figure 5 shows the results. The vertical axis plots of the differential of the invariant (which in practice corresponds very closely to a crystallization exotherm in differential scanning calorimetry), are derived from the data using an algorithm based on the method of Savitzky and Golay [28]. The four peaks indicate the maximum crystallisation rate for each of the materials. Compared to the PCL sample, the peak for PCL-DBS occurs sooner (higher temperature), as expected by increased nucleation [24], while that for PCL-PVB occurs much later (lower temperature), owing to the suppression of nucleation as also observed by optical microscopy (Figure 2b). The maximum value of the derivative is noticeably lower corresponding to a slower crystallisation rate and the peak is much broader indicating crystallisation taking place over a wider temperature range. This concurs with the observation that the invariant itself reaches a maximum about 11 °C below the point of greatest crystallisation rate, as compared with 8–9° below for all the other specimens.

## 4. Discussion

The maximum rate of crystallisation for the PCL/DBS/PVB sample is about 5 °C below that observed for the PCL sample. However this is considerably above the peak temperature for PCL/PVB. The addition of the PVB to the PCL/PVB appears to have significantly deactivated the DBS as a nucleant as measured by a shift in the maximum crystallisation temperature of about 10 °C, and this corresponds to the smaller lamellar long period indicated by the SAXS measurements. We emphasise that shear flow is imposed on the polymer melt ~40 °C above the crystallisation temperature of PCL. As the crystallisation of the sheared PCL melt reveals, at the point of crystallisation the melt itself has no memory of the shear flow. The behaviour reported above is the consequence of the distribution and nature of the nanoparticles and their influence on the nucleation of the PCL. There is the possibility that the presence of the slightly higher molecular weight PVB could alter the flow behaviour of the overall system. However, we can discount this possibility as the WAXS (Figure 2b) and SAXS (Figure 3b) patterns for the PCL/PVB sample indicate clearly an isotropic distribution of PCL crystals. Moreover, if PVB chains were significantly extended in the shear flow this would promote the development of the type of lamellar orientation observed in Figure 4c rather than that actually observed (Figure 4d).

It is more probable that the PVB acts to suppress the crystallisation of the DBS. The optical microscopy shows that this is indeed the case at least in part. The morphology shown in Figure 2d was obtained by crystallising from a melt in which the DBS was fully dissolved. From the optical specimen, it appears that once dissolved, the DBS is hindered from recrystallising by the PVB such that the morphology is similar to that of the PCL/PVB sample. However, from the presence of a significant level of small-angle scattering around the beam stop for the PCL/DBS/PVB sample (Figure 4d) we can deduce any suppression is only partial: we estimate the reduction ~50%. However, the fact that this scattering is not highly anisotropic is significant. We propose that the presence of the PVB during the preparation partially inhibits the growth of the DBS particles. This limits the length to breadth ratio of the DBS particles and modifies the behaviour of the particles in the shear flow stage of the processing cycle.

Jeffrey showed particles in a fluid during shear flow may exhibit a number of orientation states [29]. A number of studies have considered the effect of the anisotropy of the particle for example [30,31] and observations of both alignments parallel to the flow direction and to the vorticity direction have been reported for carbon nanotubes in shear polymer melts [32]. Gunes et al., have explored the effects of both the particle aspect ratio and the rheological parameters of the matrix polymer on the orientation behaviour of the particles in shear flow [33]. In materials with a low Deborah number (product of relaxation time and shear rate), the fluid elasticity can damp the particle rotations. In this study, we focus on fluid systems with higher Deborah numbers and Leal has proposed a critical shear rate for the transition from a vorticity orientation (normal to the flow) to flow axis orientation which is inversely related to the particle aspect ratio [33]. The detailed experimental study of Gunes et al., reveals a rich array of behaviour which matches theory in part.

We propose that the addition of PVB to the PCL/DBS system results in fewer and less anisotropic particles which when subjected to modest shear flow adopt an alignment preferentially parallel to the vorticity axis and normal to the flow direction. We anticipate this is principally due to the reduced aspect ratio in line with the prediction of Leal [34], but there are other consequences of the change of concentration for example, the elasticity of the system. Without the PVB, highly extended DBS particles are formed which align preferentially parallel to the flow direction in line with Leal’s prediction under the same shear rate as used in the PCL/DBS/PVB system. On cooling, these particles act to nucleate and direct the crystallisation of the PCL so that the lamellae grow normal to the surface of the particle. As a consequence, for PCL/DBS/PVB and PCL/DBS, the subsequent lamellar growth directions are very different. In both cases, the lamellae grow out radially from the DBS particles but with symmetry axes which are orthogonal to each other. We anticipate that by adjusting the proportion of the PVB introduced in to the system, we can control the inhibiting extent of the PVB on the DBS and thereby prepare samples with a range of different crystalline texture.

**Figure 6:**
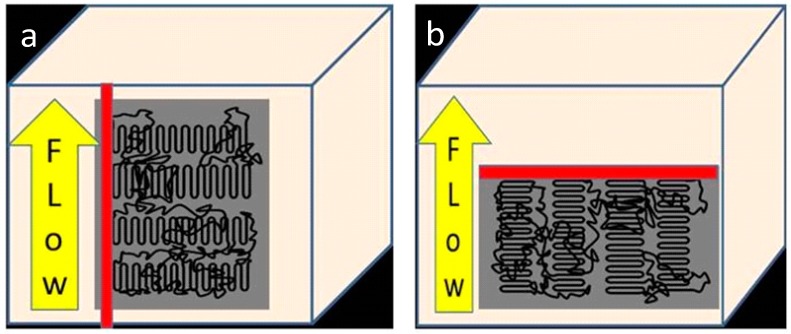
Schematic of the geometry of the templating mechanism for PCL/DBS. The red rod represents the DBS nanofibril aligned (**a**) (**left**) parallel with the flow direction and (**b**) (**right**) normal to the flow direction. This directs the growth direction of the lamellar crystals to be normal to the long axis of the DBS fibril as described in the text.

Controlling the morphology of a semi-crystalline polymer can be achieved in a variety of ways yielding major changes to properties. The addition of nucleating agents to polymers may be used to control the crystal structure as in the case of β-nucleating agents for isotactic polypropylene [35], to control of the spherulite size in packaging films [12] and in general the addition of nucleating agents leads to stiffer products [12]. We have shown in a series of papers how nucleating agents can be exploited to provide a common orientation of row nuclei to generate structures with a common lamellar crystal orientation [5,7,24]. Figure 6 shows the geometry of this process. We need to bear in mind that this nucleating effect will be cylindrically symmetric and hence any compounds diffusing from the surface of the sample in to the interior will have facile access to the amorphous regions between the crystalline lamellar. As a consequence a number of approaches have been tried to provide polymer films with lower permeability for oxygen diffusion to provide higher performance barrier films for food and medical packaging [36]. The ideal arrangement of the crystal lamellae is an arrangement in which the lamellar normal is perpendicular to the polymer film as shown in Figure 6b This provides for such an arrangement so that the lamellar crystals provide the maximum obstruction to the diffusing oxygen pathways thereby increasing tortuosity increasing the diffusion pathways [37]. PCL is a biodegradable polymer and is biocompatible. It is widely used to manufacture implants such as scaffolds for tissue engineering. The degradation of the PCL involves the diffusion of bodily fluids in to the polymer initiating hydrolytic degradation [38,39]. The same arguments relevant to gas diffusion also apply to such degradation of the morphology shown in Figure 6b will be much slower than that observed for the morphology shown in Figure 6a [39]. Of course these different morphologies will also lead to changes in mechanical properties such as stiffness due to the anisotropy of the properties of the PCL crystals.

## 5. Conclusions

When poly(ε-caprolactone) is nucleated by dibenzylidene sorbitol in the presence of a terpolymer based on polyvinyl butyral there is an orthogonal switch in the symmetry axis of the resultant lamellar orientation as compared with PCL nucleated by DBS alone.

Previously, it has been shown that the aligned extended nanoparticles formed through the use of small amounts of DBS in conjunction with modest flow fields will nucleate lamellae with their *c*-axes parallel to the particles and to the flow direction. We confirm that small quantities of a terpolymer based on polyvinyl butyral inhibit the nucleation in PCL by itself and show that its presence can also modify the behaviour of the DBS. In particular, the presence of the PVB limits the aspect ratio of the DBS particles which form and this alters the preferred orientation of the DBS particles in the sheared polymer melt from parallel to normal to the flow direction. This morphology is suitable for barrier property films and will influence the degradation rates in bioabsorbable biomedical products prepared from PCL.

## References

[1] Smith D.K. (2009). Lost in translation? Chirality effects in the self-assembly of nanostructured gel-phase materials. Chem. Soc. Rev..

[2] Sangeetha N.M., Maitra U. (2005). Supramolecular gels: Functions and uses. Chem. Soc. Rev..

[3] Weiss R.G., Terech P. (2006). Molecular Gels: Materials with Self-Assembled Fibrillar Networks.

[4] Terech P., Weiss R.G. (1997). Low Molecular Mass Gelators of Organic Liquids and the Properties of Their Gels. Chem. Rev..

[5] Nogales A., Mitchell G.R., Vaughan A.S. (2003). Anisotropic Crystallization in Polypropylene Induced by Deformation of a Nucleating Agent Network. Macromolecules.

[6] Nogales A., Olley R.H., Mitchell G.R. (2003). Directed Crystallisation of Synthetic Polymers by Low-Molar-Mass Self-Assembled Templates. Macromol. Rapid Commun..

[7] Mitchell G.R., Wangsoub S., Nogales A., Davis F.J., Olley R.H., Mitchell G.R., Tojeira A. (2016). Controlling Morphology Using Low Molar Mass Nucleators. Controlling the Morphology of Polymers: Multiple Scales of Structure and Processing.

[8] Ping Q. (2016). Polymer Morphology: Principles, Characterization, and Processing.

[9] Kimata S., Sakurai T., Nozue Y., Kasahara T., Yamaguchi N., Karina T., Shibyama M., Kornfield J.A. (2007). Report Molecular Basis of the Shish-Kebab Morphology in Polymer Crystallization. Science.

[10] Bassett D.C., Kleintjens L.A., Lemstra P.J. (1986). Lamellar Organization in Polymer Spherulites. Integration of Fundamental Polymer Science and Technology.

[11] Vaughan S., Barré L.L., Zhao Y., Sutton S.J., Swingler S.G. (2003). On additives, morphological evolution and dielectric breakdown in low density polyethylene. Eur. Polym. J..

[12] Zweifel H. (2001). Plastics Additives Handbook.

[13] Planell J.A., Best S.M., Lacroix D., Merolli A. (2009). Bone Repair Biomaterials.

[14] Kim D., Kim S.-W. (2003). Barrier property and morphology of polypropylene/polyamide blend film. Korean J. Chem. Eng..

[15] Smith T.L., Masilamani D., Bui L.-G., Khanna Y.-P., Bray R.G., Hammond W.B., Curran S., Belles J.J., Binder-Castelli S. (1994). The Mechanism of Action of Sugar Acetals as Nucleating Agents for Polypropylene. Macromolecules.

[16] Mandelkern L. (2004). Crystalization of Polymers.

[17] Thierry A., Straupé C., Wittmann J.-C., Lotz B. (2006). Organogelators and Polymer Crystallisation. Macromol. Symp..

[18] Sundararajan P.R. (2016). Physical Aspects of Polymer Self-Assembly.

[19] Keith H.D., Padden F.J., Russell T.P. (1989). Morphological changes in polyesters and polyamides induced by blending with small concentrations of polymer diluents. Macromolecules.

[20] Nozue Y., Kurita R., Hirano S., Kawasaki N., Ueno S., Iida A., Nishi T., Amemiya Y. (2003). Spatial distribution of lamella structure in PCL/PVB band spherulite investigated with microbeam small- and wide-angle X-ray scatterin. Polymer.

[21] Nozue Y., Hirano S., Kurita R., Kawasaki N., Ueno S., Iida A., Nishi T., Amemiya Y. (2004). Co-existing handednesses of lamella twisting in one spherulite observed with scanning microbeam wide-angle X-ray scattering. Polymer.

[22] Ikehara T., Jinnai H., Kaneko T., Nishioka H., Nishi T. (2007). Local lamellar structures in banded spherulites analyzed by three-dimensional electron tomography. J. Polym. Sci. Part B Polym. Phys..

[23] Nogales A., Thornley S.A., Mitchell G.R. (2004). Shear Cell for In Situ WAXS, SAXS, and SANS Experiments on Polymer Melts Under Flow Fields. J. Macrom. Sci. Phys..

[24] Wangsoub S., Oiley R.H., Mitchell G.R. (2005). Directed Crystallisation of Poly(ε-caprolactone) using a Low-Molar-Mass Self-Assembled Template. Macromol. Chem. Phys..

[25] Towns-Andrews E., Berry A., Bordas J., Mant G.R., Murray P.K., Roberts K., Sumner I., Worgan J.S., Lewis R., Gabriel A. (1989). Time-resolved x-ray diffraction station: X-ray optics, detectors, and data acquisition. Rev. Sci. Instrum..

[26] Mohan S., Olley R.H., Vaughan A.S., Mitchell G.R., Mitchell G.R., Tojeira A. (2016). Evaluating Scales of Structure in Polymers. Controlling Controlling the Morphology of Polymers: Multiple Scales of Structure and Processing.

[27] Roe R.-J. (2000). Methods of X-ray and Neutron Scattering in Polymer Science.

[28] Savitzky A., Golay M.J.E. (1964). Smoothing and Differentiation of Data by Simplified Least Squares Procedures. Anal. Chem..

[29] Jeffreys G.B. (1922). The motion of ellipsoidal particles immersed in a viscous fluid. Proc. R. Soc. Lond..

[30] Iso Y., Koch D.L., Cohen C. (1996). Orientation in simple shear flow of semi-dilute fiber suspensions 1. Weakly elastic fluids. J. Non-Newton. Fluid Mech..

[31] Iso Y., Cohen C., Koch D.L. (1996). Orientation in simple shear flow of semi-dilute fiber suspensions 2. Highly elastic fluids. J. Non-Newton. Fluid Mech..

[32] Hobbie E.K., Wang H., Kim H., Lin-Gibson S., Grulke S. (2003). Orientation of carbon nanotubes in a sheared polymer melt. Phys. Fluids.

[33] Gubes D.Z., Scirocco R., Mewis J., Vermant J. (2008). Flow-induced orientation of non-spherical particles: Effect of aspect ratio and medium rheology. J. Non-Newton. Fluid Mech..

[34] Leal L.G. (1975). The slow motion of slender rod-like particles in a second-order fluid. J. Fluid Mech..

[35] Papageorgiou D.G., Chrissafis K., Bikiaris D.N. (2015). β-Nucleated Polypropylene: Processing, Properties and Nanocomposites. Polym. Rev..

[36] Mitchell G.R., Silvestre C., Cimmino S. (2013). Characterisation of safe nanostructured polymers. Ecosustainable Polymer Nanomaterials for Food Packaging.

[37] Bharadwaj R.K. (2001). Modeling the Barrier Properties of Polymer-Layered Silicate Nanocomposites. Macromolecules.

[38] Lam C.X., Savalani M.M., Teoh S.H., Hutmacher D.W. (2008). Dynamics of in vitro polymer degradation of polycaprolactone-based scaffolds: Accelerated versus simulated physiological conditions. Biomed. Mater..

[39] Mitchell G.R., Davis F.J., Olley R.H., Mitchell G.R., Tojeira A. (2016). Scales of Structure in Polymers. Controlling the Morphology of Polymers: Multiple Scales of Structure and Processing.

